# Inferring antenatal care visit timing in low- and middle-income countries: Methods to inform potential maternal vaccine coverage

**DOI:** 10.1371/journal.pone.0237718

**Published:** 2020-08-20

**Authors:** Ranju Baral, Jessica Fleming, Sadaf Khan, Deborah Higgins, Nathaniel Hendrix, Clint Pecenka

**Affiliations:** 1 PATH, Seattle, WA, United States of America; 2 The Comparative Health Outcomes, Policy, and Economics (CHOICE) Institute, University of Washington, Seattle, WA, United States of America; Boston University School of Public Health, UNITED STATES

## Abstract

**Background:**

The timing of antenatal care (ANC) visits directly affect health intervention coverage and impact, especially for those interventions requiring strict gestational age windows for administration, such as maternal respiratory syncytial virus (RSV) vaccine. Existing nationally representative population-based surveys do not record the timing of ANC visits beyond the first, limiting the availability of reliable data around timing of subsequent ANC visits in most low- and middle-income countries (LMICs). Here, we describe a model that estimates the timing of ANC visits by gestational age using publicly available multi-country survey data.

**Methods and findings:**

We used the Demographic and Health Surveys (DHS) data from 69 LMICs. We used several factors to estimate the timing of subsequent ANC visits by gestation age: the timing of the first ANC visit (ANC1) in a given pregnancy, derived from the DHS; the country’s reported average ANC coverage at each ANC visit (ANC1 through the fourth ANC visit [ANC4]); and the World Health Organization’s guidance on recommended ANC visit. We then used the timing of ANC visit by gestation age to predict the coverage of a potential maternal RSV vaccine administered at 24–36 weeks of gestation. We calculated the maternal immunization coverage by summing the number of eligible women vaccinated at any ANC visit divided by the total number of pregnant women. We find, in general, countries with higher ANC1 coverage were predicted to have higher vaccination coverage. In 82% of countries, the modeled vaccine coverage is less than ANC4 coverage.

**Conclusions:**

The methods illustrated in this paper have implications on the precision of estimating impact and programmatic feasibility of time-critical interventions, especially for pregnant women. The methods can be easily adapted to vaccine demand forecasts models, vaccine impact assessments, and cost-effectiveness analyses and can be adapted to other maternal interventions that have administration timing restrictions.

## Introduction

Improvements in health services and preventive health interventions such as immunization have been critical in reducing neonatal and infant mortality and providing broader health improvement over the past several decades [1]. With many causes of neonatal mortality rooted in the antenatal period, antenatal care (ANC) provides an opportunity to provide preventive and promotive health care to improve health outcomes for both mothers and their neonates. Given the centrality of ANC as an access point for pregnant women, ANC visits are a potentially promising context in which to deliver maternal vaccines and other services for pregnant women.

A number of vaccines are currently given safely and effectively during pregnancy. Tetanus toxoid-containing vaccines (TTCV) are the most common, and influenza and pertussis vaccines are used in some settings. Additional maternal vaccines are in development, including for respiratory syncytial virus (RSV) and group B streptococcus. And while some maternal vaccines, like TTCV, have no restrictions on when they are given, others need to be administered during specific gestational age windows to be most effective. For example, the World Health Organization (WHO) preferred product characteristics for RSV vaccines recommend administration in the second or third trimester of pregnancy [2].

If ANC is the anticipated delivery platform for maternal vaccines that have restricted administration windows, estimates of ANC visit timing will directly affect vaccine coverage estimates and other interventions provided through ANC that require strict gestational age windows of administration.

Limited data on ANC timing is available through the Demographic Health Surveys (DHS) and Multiple Indicator Cluster Surveys (MICS) [3–5]. Such surveys are conducted in many low- and middle-income countries (LMICs) and provide standardized health data across countries. For instance, DHS records the number of ANC visits attended, services provided at these visits, and the timing (estimated gestational age) of the initial ANC visit. However, the survey does not include information on the timing of subsequent ANC visits for women who had more than one antenatal contact.

The purpose of this paper is to demonstrate a method to estimate ANC attendance by gestational age in LMICs and use it to estimate coverage of a maternal RSV vaccine. We use a maternal RSV vaccine as an example as it is delivered during a specific time period during pregnancy and the lack of a clear vaccine coverage proxy was a challenge in other analyses projecting the impact and cost-effectiveness of maternal RSV vaccination. This method can be applied to vaccine demand forecasts models, vaccine impact assessments, and cost-effectiveness analyses and can be adapted to other maternal interventions that have administration timing restrictions.

## Methods

To estimate the timing of ANC visits in LMICs by gestational age, we used the most recent DHS data available from 69 LMICs, published between 2001 and 2018 (countries listed in S1 Appendix).

We used the timing of the first ANC visit (ANC1) in a given pregnancy, derived from the DHS; the country’s reported average ANC coverage at each ANC visit (ANC1 through the fourth ANC visit [ANC4]); and WHO’s focused antenatal care (FANC) guidance on recommended ANC visit timing to predict the timing of subsequent ANC visits among pregnant women. These variables were inputted into a model to estimate the coverage of a single dose of maternal RSV vaccine administered at 24–36 weeks of gestation.

We also compared our estimates of RSV maternal immunization coverage to ANC1, ANC4, and protection at birth (PAB) for TTCV. PAB, which accounts for the number of TTCV doses previously received by the mother, the interval between doses, and the time since her last dose (using card or verbal history), is often discussed as a comparable metric for assessing maternal immunization coverage [6,7].

### Data

The definition of the model variables and how they were incorporated into the analysis are described below.

#### ANC 1 timing

DHS are nationally representative cross-sectional surveys with relatively large sample sizes (usually 5,000–30,000 households per country) conducted approximately every 3 to 5 years across many LMICs [3]. Standard questionnaire modules are provided for countries to adapt to their local contexts. The maternal health module gathers individual-level information on indicators such as number of ANC visits made in recent pregnancies, the timing of ANC1, and the services received during the visit [8].

We extracted data from DHS country surveys regarding timing of ANC1 attendance from each pregnant woman surveyed (DHS questionnaire section 4, question 411) for all births reported in or after 2000 in study countries. The most recent data available were used for countries with multiple surveys.

#### ANC coverage

ANC1 and ANC4 coverage were extracted from a United Nations Children's Fund (UNICEF) database, using the most recent data available [9]. The UNICEF database sources ANC coverage data mostly from DHS and other national-level surveys, providing up-to-date coverage estimates across countries. ANC1 coverage includes country-specific percentage of pregnant women who attended at least one ANC visit during their most recent pregnancy. ANC4 coverage includes the percentage of pregnant women who attended four or more ANC visits during their most recent pregnancy. Coverage of the second and third ANC visits (ANC2 and ANC3, respectively) were inferred using the estimates of ANC1 and ANC4 coverage, assuming a linear decrease in coverage between the ANC1 and ANC4 visits over each successive visit.

#### Service availability and acceptance proxy

Most DHS surveys include data on ANC visitors who reported receiving basic services during any ANC visit in their last pregnancy. This basic set of ANC services include provision of information about any pregnancy complications; patient measurements, including weight, height, and blood pressure; urine and blood sample collection; and iron supplementation. We calculated the average reported number of services received during ANC visits and used it as a proxy for service availability and acceptance in each country. The service availability and acceptance proxy was subsequently applied to RSV vaccine coverage (described below) to account for availability and acceptance of services among pregnant women at a given ANC visit.

#### Eligible population for vaccination

We estimated the total number of pregnant women by country by adding the stillbirth estimate of the country to the annual live births for the year 2018 reported in the World Population Prospects Data [10,11]. All pregnant women who attended an ANC visit within the recommended gestational window for receipt of maternal RSV vaccine (24–36 weeks) were considered eligible for vaccination.

### Estimating timing of ANC attendance by gestation week

The DHS reports ANC1 visit timing by gestation month. We assumed each month was composed of exactly four full weeks, and that visits were distributed evenly across the weeks. For example, for all pregnant women who reported attending ANC1 in their second month of pregnancy, we assigned one quarter each of those visits to gestational weeks 5, 6, 7, and 8.

All pregnant women were assumed eligible for an ANC1 visit. To generate the eligible population by gestation week, we multiplied the total number of pregnant women by the percent of ANC1 visits per gestation week. This value was multiplied by the percent ANC1 coverage to estimate ANC1 attendees by gestation week.

We estimated the timing of subsequent ANC visits using the gap between visits per the FANC ANC recommendations. The FANC guidance recommends ANC1 at 8–12 weeks and ANC2 at 24–26 weeks. To estimate timing between ANC1 and ANC2 visits, we subtracted the upper bound of ANC1 (12 weeks) from the upper bound of ANC2 (26 weeks), arriving at 14 weeks between these visits. We assumed that women returning for ANC2 were distributed across the recommended window and assigned 50 percent to 14 weeks after ANC1; 25 percent to 13 weeks after ANC1; and 25 percent to 15 weeks after ANC1. As per the gap established by the FANC guidance, women attending ANC2 were assumed to return for ANC3 4 weeks after their ANC2 visit, and women attending ANC3 were assumed to return for ANC4 4 weeks after their ANC3 visit. We made the same assumptions regarding distributing subsequent visits across contiguous weeks.

The share of eligible women attending a subsequent ANC visit was based on their country’s ANC coverage for the corresponding visit. Women who did not attend a given ANC visit and women reported as having attended an ANC visit after 40 weeks of gestation were considered out of range and ineligible for subsequent ANC visits.

### Estimating RSV maternal immunization coverage (Table 1)

All pregnant women who attended an ANC visit between 24 and 36 weeks of gestation were assumed eligible to receive RSV vaccine until they were classified as vaccinated. Vaccination coverage by ANC visit was calculated by multiplying the number of unvaccinated women attending an ANC visit between 24 and 36 weeks of gestation by the service availability and acceptance proxy. Unvaccinated women in a given visit were considered eligible for vaccination during subsequent ANC visits. We calculated the overall maternal immunization coverage for a country by summing the number of eligible women vaccinated at any ANC visit divided by the total number of pregnant women.

**Table 1:** Estimating maternal vaccination coverage by ANC visit.

ANC visit	Women eligible for vaccination	Eligible women who visited ANC in a given window	Eligible women who received vaccination	Women ineligible to receive vaccine at next visit
**ANC1**	All pregnant women	% pregnant women attending ANC1 during vaccination window * **A** * % ANC1 coverage	**B** * Service availability and acceptance proxy	Women who reached 40 weeks’ gestation or greater by the subsequent visit + **C**
	**= [A]**	**= [B]**	**= [C]**	**= [D]**
**ANC2**	Number of pregnant women eligible for ANC2, i.e., (**A–D**)	**E** * % ANC2 coverage	**F** * Service availability and acceptance proxy	Women who reached 40 weeks’ gestation or greater by the subsequent visit + **G**
	**= [E]**	**= [F]**	**= [G]**	**= [H]**
**ANC3**	Number of pregnant women eligible for ANC3, i.e., (**E–H**)	**I** * % ANC3 coverage	**J** * Service availability and acceptance proxy	Women who reached 40 weeks’ gestation or greater by the subsequent visit + **K**
	**= [I]**	**= [J]**	**= [K]**	**= [L]**
**ANC4**	Number of pregnant women eligible for ANC3, i.e., (**I–L**)	**M** * % ANC4 coverage	**N** * Service availability and acceptance proxy	Women who reached 40 weeks’ gestation or greater by the subsequent visit + **O**
	**= [M]**	**= [N]**	**= [O]**	**= [P]**

$ANC\;attendance\;in\;a\;given\;window=\frac{\left[B]+[F]+[J]+[N\right]}{\left[A\right]}$

$Overall\;maternal\;vaccination\;coverage=\frac{\left[C]+[G]+[K]+[O\right]}{\left[A\right]}$

"ANC attendance in a given window” counts women more than once if their ANC visit occurs multiple times within the window.

## Results

DHS surveys from 69 countries, published between 2001 and 2018, were publicly available as of June 2019. One country, Bangladesh, did not include ANC timing data and was excluded from the analysis. Of the DHS datasets used in this paper, about 37 percent (25/68) were from year 2015–2018 and more than 80 percent (56/68) were from after 2010. More than 50 percent of the countries included in this study were from the sub-Saharan Africa region. Across study countries, median ANC1 attendance was 93.3 percent (range 54.7 to 99.6 percent); median ANC4 attendance was 66.4 percent (range 17.8 to 96.0 percent). A full list of countries and the DHS versions used in the model are included in S1 Appendix.

### Timing of ANC attendance by gestation period

**Fig 1:**
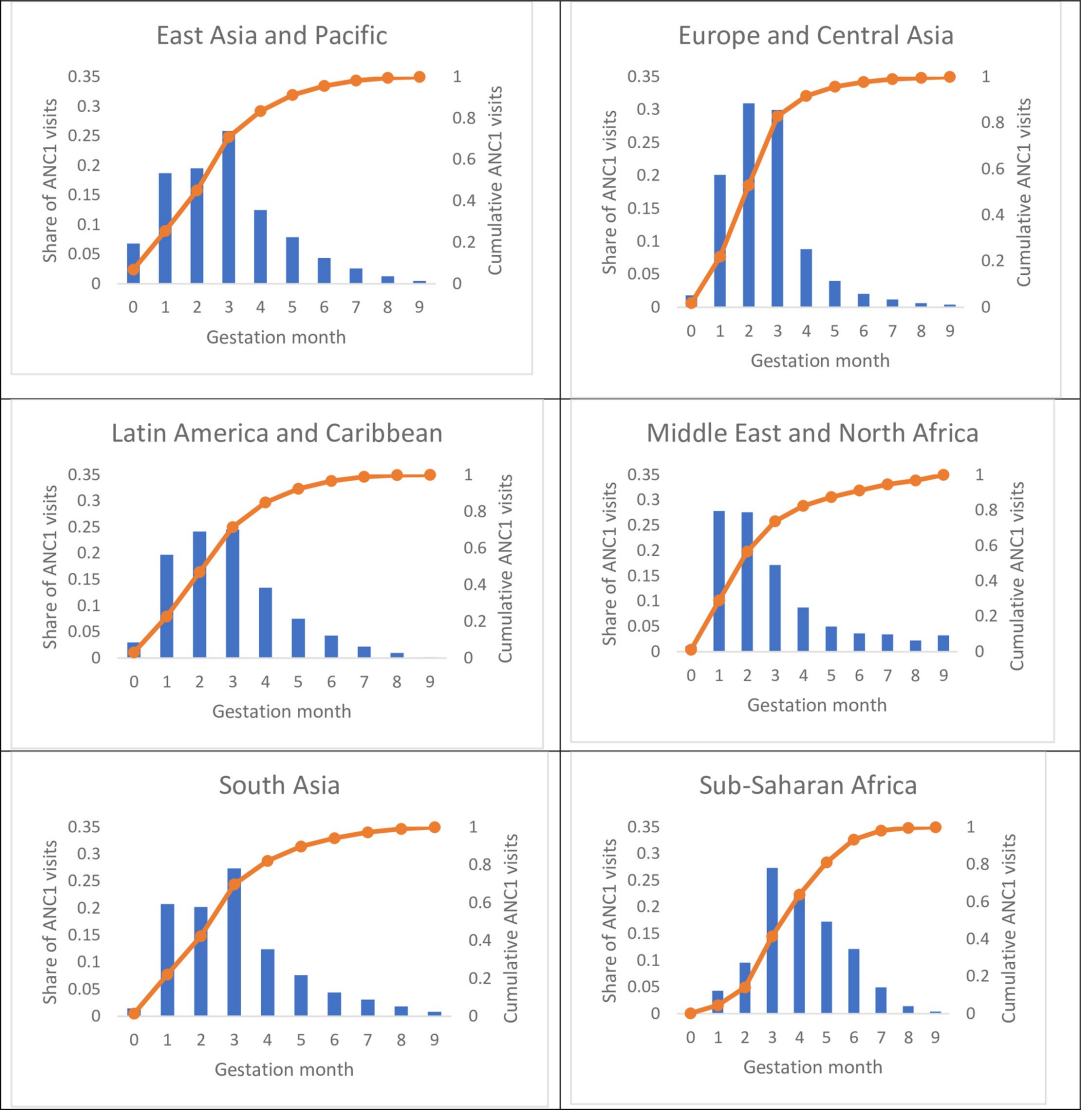
ANC1 timing by gestation month by geographic region.

Fig 1 depicts the average timing of ANC1 visits by gestational age and geographic region as reported in the DHS. More than 50 percent of ANC1 visits occurred within the first 3 months of pregnancy in two regions: Europe and Central Asia, and the Middle East and North Africa. A plurality of women (42, 45, and 47 percent) attended ANC1 within the first 3 months of pregnancy in South Asia, East Asia and Pacific, and Latin America and Caribbean regions. In contrast, the ANC1 visit typically occurred later in pregnancy for women in the sub-Saharan Africa region, where only 14 percent of ANC1 attendance was reported to occur within the first 3 months of pregnancy.

**Fig 2:**
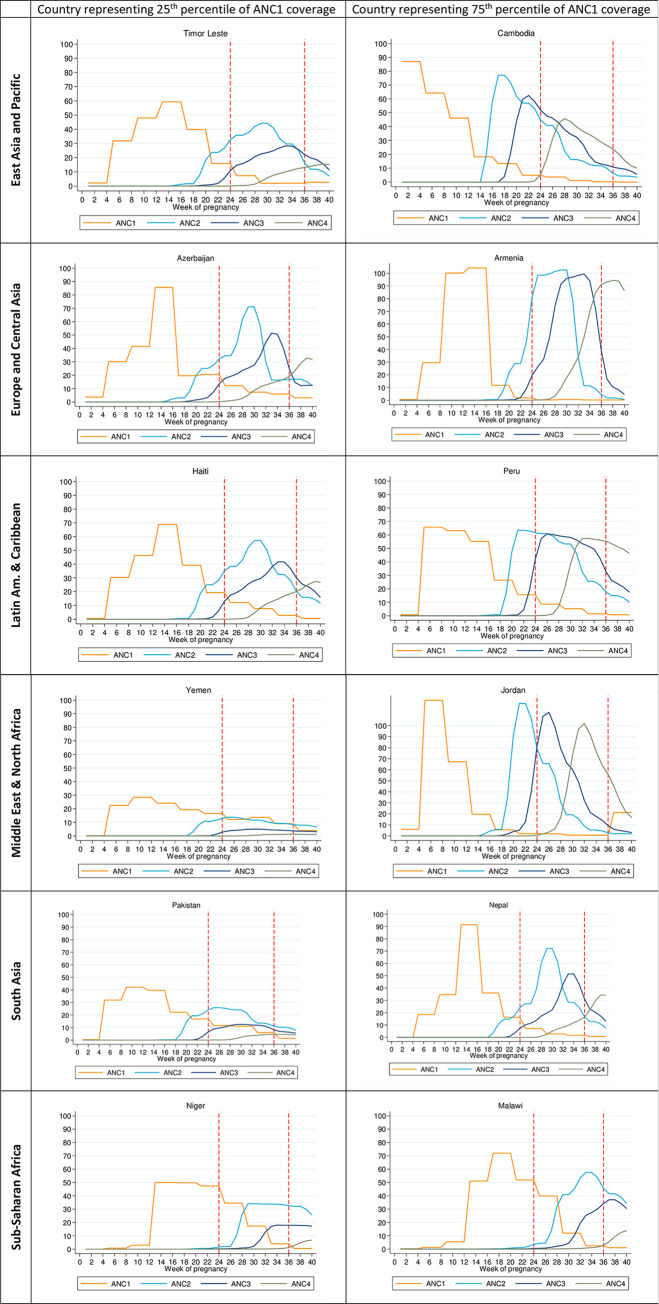
ANC visits and maternal RSV vaccine eligibility by gestation week in example countries representing 25^th^ and 75^th^ percentile ANC1 coverage by geographic region. Figures in the left column represent countries with ANC1 coverage at the twenty-fifth percentile for the region, and figures in the right column represent countries at the seventy-fifth percentile ANC1 coverage for the region. The two horizontal lines (in red) indicate the anticipated RSV vaccination window.

The average timing of ANC visits varied widely by country (see S2 Appendix). In Fig 2, we depict the reported timing for each ANC visit in two example countries, representing the 25th and 75th percentile of ANC1 coverage in their respective regions. In each graph, the area under the curve represents the number of pregnant women per 1,000 attending a given ANC visit. We also show how the number of women attending ANC visits compares with a vaccination window of 24–36 weeks, represented by the red dotted lines, across the example countries.

### RSV maternal immunization coverage

**Fig 3:**
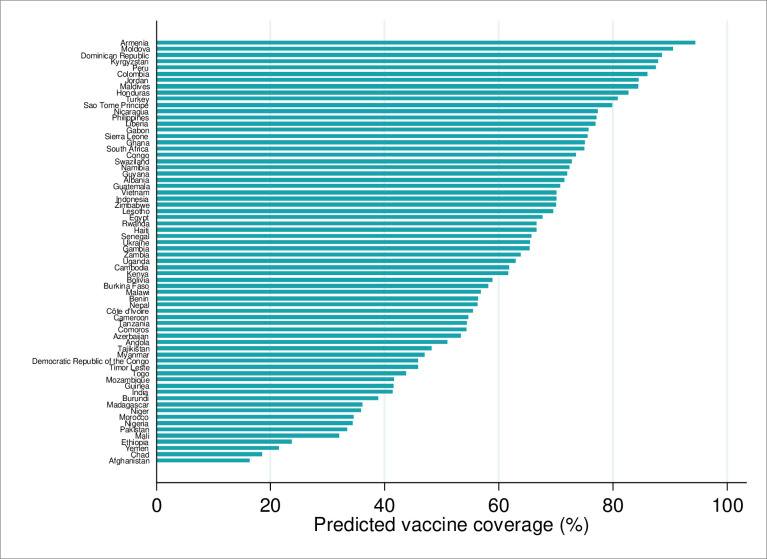
Predicted RSV maternal immunization coverage at 24–36 weeks gestation by country.

Our predictions of RSV maternal immunization coverage at 24–36 weeks of gestation by country, adjusted for the service availability and acceptance proxy, are illustrated in Fig 3. The service availability and acceptance proxy estimates ranged from 40.8 percent to 97.4 percent (median 82.5 percent) across study countries (see S3 Appendix). In general, countries with higher reported ANC4 coverage had higher service availability and coverage estimates (see S4 Appendix). Across all study countries, our median vaccine coverage estimate was 65 percent (range 16 to 94 percent). Four countries, all classified as low income by the World Bank (Afghanistan, Chad, Yemen, and Ethiopia), had the lowest predicted vaccine coverage (< 25 percent). Armenia (upper-middle income) and Moldova (lower-middle income) had the highest vaccine coverage (> 90 percent). Eleven of the 68 study countries were predicted to have > 80 percent vaccine coverage.

**Fig 4:**
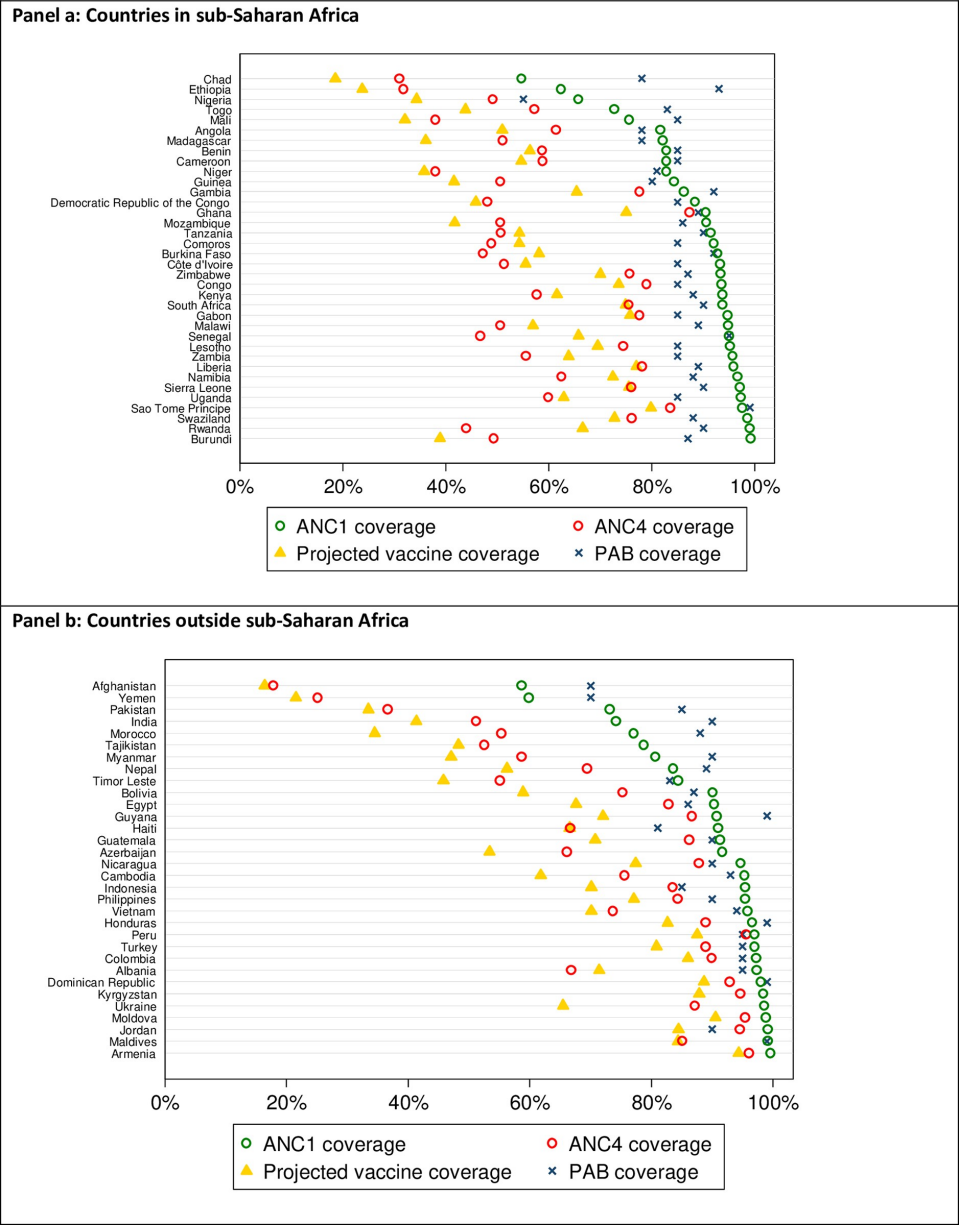
Comparison of RSV maternal immunization coverage predictions with estimates of ANC1, ANC4, and PAB.

As a validation exercise, we compared our predicted RSV maternal immunization coverage with reported ANC1 and ANC4 coverage estimates [9] and PAB [6] estimates against tetanus by country (Fig 4). ANC1 coverage estimates are higher than modeled vaccine coverage in all countries. In most countries (56/68 countries; 82 percent), our modeled vaccine coverage is less than ANC4 coverage estimates. Excluding six countries without available PAB estimates, the PAB estimates are higher than the vaccine coverage estimates in all countries. In general, countries with higher ANC1 coverage and PAB estimates were predicted to have higher vaccination coverage. Across countries, the average difference between the vaccination coverage estimates and ANC1 coverage is 28 percent (5 to 60 percent); between vaccination coverage estimates and ANC4 coverage is 5 percent (–23 to 22 percent); and between vaccination coverage and PAB is 28 percent (6 to 69 percent).

## Discussion

Understanding the timing of ANC visits is critical for providing maternal health interventions tied to specific gestational age windows that maximize their utility and effectiveness. Existing nationally representative population-based surveys do not record the timing of ANC visits beyond the first, limiting the availability of reliable data around timing of subsequent ANC visits in most LMICs. Here we describe a model that estimates the timing of subsequent ANC visits by gestational age using publicly available DHS data. We applied our model to estimate the coverage of a potential maternal RSV vaccine provided to pregnant women during routine ANC visits.

As a crude validation, we compared our RSV maternal immunization coverage estimates to PAB and ANC1 and ANC4 coverage, as they are the closest metrics commonly used in the literature to measure maternal health intervention coverage. Across most countries, ANC1 coverage is often the highest, followed by PAB coverage, ANC4 coverage, and our estimate of immunization coverage (Fig 4). In countries with comparatively low ANC coverage (both ANC1 and ANC4), PAB estimates can exceed ANC1 coverage. This may be expected since PAB estimates account for tetanus vaccine received during pregnancy as well as previous protective doses. Further, TTCV can be administered at any time during pregnancy. Comparing these estimates, therefore, does not directly assess the validity of the methods developed here for maternal RSV vaccine coverage. Nonetheless, looking at these estimates together provides context and opportunity to evaluate the robustness of our projections.

In addition to illustrating methods for estimating maternal vaccine coverage and comparing our estimates with other potential indicators, this study provides initial insight into factors that might constrain or enhance maternal immunization coverage. Even in countries with high ANC coverage, administration of interventions that require a strict gestational age window can be compromised if the first ANC visit occurs too late in pregnancy, if ANC visits are infrequent or delayed, or if services are not available or acceptable to women during an ANC visit. We found that each of these variables can impact immunization coverage estimates in a variety of ways. For example, Burundi and Rwanda have relatively high and comparable ANC1 and ANC4 coverage, and in both countries, women start ANC1 relatively early—before 3 months in pregnancy (47 percent Burundi, 56 percent Rwanda). Their immunization coverage estimates, however, differ by more than 30 percent points, driven primarily by a difference in their service availability and acceptance proxy. Conversely, Burundi and Afghanistan both have relatively comparable proportions of ANC1 attendees in the first 3 months (~47 percent) and similar service availability and acceptance (51 percent in Afghanistan, 41 percent in Burundi), but Afghanistan’s lower overall ANC1 and ANC4 coverage drives its lower immunization coverage estimate.

Our study has several limitations. We did not calibrate DHS data to account for temporal differences in survey year across counties. The DHS surveys also did not cover the same year across all countries; we used the most recent DHS data available for each. Further, we applied the latest ANC coverage estimates available for each country to estimated 2018 populations of pregnant women. Assuming temporal improvement in ANC timing as well as coverage, our estimates of ANC attendance and vaccine coverage may be conservative. We also applied the service availability and acceptance proxy to each ANC visit, although the DHS surveys measure coverage of basic ANC services for all visits combined, and some of the interventions included in the basic service package are offered at multiple visits. Further, our service availability and acceptance proxy were only moderately correlated with PAB (r = 0.47), suggesting that some countries may choose to prioritize vaccination over other basic services such as measuring height and collecting urine samples that make up the proxy indicator. Applying this discounted service availability and acceptance proxy variable produces conservative vaccination coverage estimates.

The WHO’s 2016 ANC guidelines include twice the number of visits compared to the FANC guidelines, four of which fall within the optimal vaccination administration window for the illustrative maternal RSV vaccine. The four ANC visits and the timing for subsequent visits as suggested by the FANC model were used in this paper as they reflect the current realities since countries have not fully incorporated updated guidance. This may change, however, as more countries adopt the new guidelines.

Although some data around timing of subsequent visits are available for Demographic Surveillance Systems, including INDEPTH [12] for a handful of countries, there is lack of a comprehensive database available across LMICs. Systematic data collection efforts on ANC visit timing is essential as time-sensitive maternal and neonatal health interventions, such as maternal immunization, gain prominence. Meanwhile, in the absence of robust data, our method demonstrates how publicly available data can be used to infer insights into ANC visit timing among pregnant women across countries. Ultimately, the methods and estimates presented in this paper may hold the greatest value in the period before generalizable empirical estimates are available.

While we demonstrate the application of our methods to vaccination coverage, this method can be adapted for other interventions by changing model parameters such as administration windows, per the requirements for that specific intervention. As countries expand the menu of services offered within the ANC visit, the model presented here can provide a framework for planning and logistics of interventions that need to be completed within specific gestational windows. These include targeted information, education, and communication messaging; delivering other maternal vaccines; diagnostic testing; repeat sonography; and initial and repeat laboratory screenings [13,14].

Success of any health intervention, in terms of conveying the health impact and effectiveness, largely hinges on the extent of its coverage among the population it purports to serve. When considering global policy and financing of any new interventions, WHO; Gavi, the Vaccine Alliance; and country decision-makers extensively evaluate the expected impact of such interventions. The methods illustrated in this paper have implications on the precision of estimating impact and programmatic feasibility of time-critical interventions, especially for pregnant women. The methods demonstrated here can easily be adapted for other interventions by changing model parameters for targeted intervention delivery.

## Conclusions

Advances in maternal and neonatal health in the coming decades will rely on improvements in routine health services, such as ANC, and the introduction and timely use of interventions like maternal vaccines. Our model is the first known attempt to estimate coverage of a maternal vaccine using existing global survey data that accounts for ANC visit timing by gestational age and service availability and acceptance. Accurate coverage estimates of maternal health interventions will become increasingly important as new maternal interventions become available and countries and donors with limited resources are required to prioritize interventions based on expected impact and operational feasibility.

## Supporting information

S1 Appendix DHS surveys available by year [3]. (DOCX)

S2 Appendix ANC1 timing by gestation month across countries [3]. (DOCX)

S3 Appendix ANC coverage and service availability and acceptance proxy estimates. (DOCX)

S4 Appendix Estimates of service availability and acceptance proxy by country used to adjust immunization coverage. (DOCX)

S5 Appendix 1: ANC1 timing by gestation month by geographic region (Source: DHS, includes data from 2010–2018 only, N = 56). 2: ANC1 timing by gestation month by geographic region (Source: DHS, includes data from 2015–2018 only, N = 25).
(DOCX)

S6 Appendix 1: Predicted RSV maternal immunization coverage at 24–36 weeks gestation by country (Includes data from 2010–2018 only, N = 56). 2: Predicted RSV maternal immunization coverage at 24–36 weeks gestation by country (Includes data from 2015–2018 only, N = 25).
(DOCX)

S7 Appendix 1 Comparison of RSV maternal immunization coverage predictions with estimates of ANC1, ANC4, and PAB (Includes data from 2010–2018 only). 2 Comparison of RSV maternal immunization coverage predictions with estimates of ANC1, ANC4, and PAB (Includes data from 2015–2018 only).
(DOCX)
