# Supplementary material for: Inferring antenatal care visit timing in low- and middle-income countries: Methods to inform potential maternal vaccine coverage
Source: PLoS One. 2020 Aug 20;15(8):e0237718. doi: 10.1371/journal.pone.0237718 (PMC7446781; doi:10.1371/journal.pone.0237718)
Supplement: S1 Appendix — (DOCX) [file pone.0237718.s001.docx]

**Appendix 1: DHS surveys available by year [3]**

| **Country** | **Geographic region** | **World Bank income group (2018)** | **DHS Dataset** | **DHS year** | **DHS version** |
| --- | --- | --- | --- | --- | --- |
| Afghanistan | South Asia | Low income | AFBR70FL | 2016 | DHS VII |
| Albania | Europe & Central Asia | Upper middle income | ALBR71FL.DTA | 2018 | DHS VII |
| Angola | Sub-Saharan Africa | Lower middle income | AOBR71FL | 2016 | DHS VII |
| Armenia | Europe & Central Asia | Upper middle income | AMBR72FL.DTA | 2016 | DHS VII |
| Azerbaijan | Europe & Central Asia | Upper middle income | AZBR52FL.DTA | 2006 | DHS V |
| Bangladesh* | South Asia | Lower middle income | BDBR72FL | 2014 | DHS VI |
| Benin | Sub-Saharan Africa | Low income | BJBR71FL.DTA | 2018 | DHS VII |
| Bolivia | Latin America & Caribbean | Lower middle income | BOBR51FL.DTA | 2008 | DHS V |
| Burkina Faso | Sub-Saharan Africa | Low income | BFBR62FL.DTA | 2010 | DHS VII |
| Burundi | Sub-Saharan Africa | Low income | BUBR70FL.DTA | 2016 | DHS VII |
| Cambodia | East Asia & Pacific | Lower middle income | KHBR73FL | 2014 | DHS VII |
| Cameroon | Sub-Saharan Africa | Lower middle income | CMBR61FL.DTA | 2011 | DHS VI |
| Chad | Sub-Saharan Africa | Low income | TDBR71FL.DTA | 2014 | DHS VII |
| Colombia | Latin America & Caribbean | Upper middle income | COBR72FL.DTA | 2015 | DHS VII |
| Comoros | Sub-Saharan Africa | Low income | KMBR61FL.DTA | 2012 | DHS VI |
| Congo | Sub-Saharan Africa | Lower middle income | CGBR60FL.DTA | 2011 | DHS VI |
| Côte d'Ivoire | Sub-Saharan Africa | Lower middle income | CIBR62FL.DTA | 2012 | DHS VI |
| Dem. Rep. of Congo | Sub-Saharan Africa | Low income | CDBR61FL.DTA | 2014 | DHS VI |
| Dominican Republic | Latin America & Caribbean | Upper middle income | DRBR61FL.DTA | 2013 | DHS VI |
| Egypt | Middle East & North Africa | Lower middle income | EGBR61FL.DTA | 2014 | DHS VI |
| Ethiopia | Sub-Saharan Africa | Low income | ETBR70FL.DTA | 2008 | DHS VI |
| Gabon | Sub-Saharan Africa | Upper middle income | GABR60FL.DTA | 2012 | DHS VI |
| Gambia | Sub-Saharan Africa | Low income | GMBR60FL.DTA | 2013 | DHS VI |
| Ghana | Sub-Saharan Africa | Lower middle income | GHBR72FL.DTA | 2014 | DHS VII |
| Guatemala | Latin America & Caribbean | Upper middle income | GUBR71FL.DTA | 2015 | DHS VI |
| Guinea | Sub-Saharan Africa | Low income | GNBR62DT.ZIP | 2012 | DHS VI |
| Guyana | Latin America & Caribbean | Upper middle income | GYBR5IFL.DTA | 2009 | DHS V |
| Haiti | Latin America & Caribbean | Low income | HTBR70FL.DTA | 2017 | DHS VII |
| Honduras | Latin America & Caribbean | Lower middle income | HNBR62FL.DTA | 2012 | DHS VI |
| India | South Asia | Lower middle income | IABR74FL | 2015 | DHS VII |
| Indonesia | East Asia & Pacific | Lower middle income | IDBR71FL | 2017 | DHS VII |
| Jordan | Middle East & North Africa | Upper middle income | JOBR71FL.DTA | 2017 | DHS VII |
| Kenya | Sub-Saharan Africa | Lower middle income | KEBR71FL.DTA | 2014 | DHS VII |
| Kyrgyzstan | Europe & Central Asia | Lower middle income | KYBR61FL.DTA | 2012 | DHS VI |
| Lesotho | Sub-Saharan Africa | Lower middle income | LSBR71FL.DTA | 2014 | DHS VII |
| Liberia | Sub-Saharan Africa | Low income | LBBR6AFL.DTA | 2013 | DHS VI |
| Madagascar | Sub-Saharan Africa | Low income | MDBR51FL.DTA | 2008 | DHS V |
| Malawi | Sub-Saharan Africa | Low income | MWBR7HFL.DTA | 2016 | DHS VII |
| Maldives | South Asia | Upper middle income | MVBR71FL | 2016 | DHS VII |
| Mali | Sub-Saharan Africa | Low income | MLBR6AFL.DTA | 2012 | DHS VI |
| Morocco | Middle East & North Africa | Lower middle income | MABR43FL | 2004 | DHS IV |
| Mozambique | Sub-Saharan Africa | Low income | MZBR62FL.DTA | 2011 | DHS VI |
| Myanmar | East Asia & Pacific | Lower middle income | MMBR71FL | 2016 | DHS VII |
| Namibia | Sub-Saharan Africa | Upper middle income | NMBR61FL.DTA | 2013 | DHS VI |
| Nepal | South Asia | Low income | NPBR7HFL | 2016 | DHS VII |
| Nicaragua | Latin America & Caribbean | Lower middle income | NCBR41FL.dta | 2001 | DHS IV |
| Niger | Sub-Saharan Africa | Low income | NIBR61FL.DTA | 2012 | DHS VI |
| Nigeria | Sub-Saharan Africa | Lower middle income | NGBR6AFL.DTA | 2013 | DHS VI |
| Pakistan | South Asia | Lower middle income | PKBR71FL.DTA | 2018 | DHS VII |
| Peru | Latin America & Caribbean | Upper middle income | PEBR6IFL.DTA | 2012 | DHS VI |
| Philippines | East Asia & Pacific | Lower middle income | PHBR70FL.DTA | 2017 | DHS VII |
| Republic of Moldova | Europe & Central Asia | Lower middle income | MBBR53FL.DTA | 2005 | DHS IV |
| Rwanda | Sub-Saharan Africa | Low income | RWBR70FL.DTA | 2014 | DHS VII |
| Sao Tome & Principe | Sub-Saharan Africa | Lower middle income | STBR50FL.DTA | 2008 | DHS V |
| Senegal | Sub-Saharan Africa | Low income | SNBR7ZFL.DTA | 2017 | DHS VII |
| Sierra Leone | Sub-Saharan Africa | Low income | SLBR61FL.DTA | 2013 | DHS VI |
| South Africa | Sub-Saharan Africa | Upper middle income | ZABR71FL.DTA | 2016 | DHS VII |
| Swaziland | Sub-Saharan Africa | Lower middle income | SZBR51FL.DTA | 2006 | DHS V |
| Tajikistan | Europe & Central Asia | Low income | TJBR71FL.DTA | 2017 | DHS VII |
| Timor-Leste | East Asia & Pacific | Lower middle income | TLBR71FL.DTA | 2016 | DHS VII |
| Togo | Sub-Saharan Africa | Low income | TGBR61FL.DTA | 2013 | DHS VI |
| Turkey | Europe & Central Asia | Upper middle income | TRBR61FL.DTA | 2013 | DHS VI |
| Uganda | Sub-Saharan Africa | Low income | UGBR7BFL.DTA | 2016 | DHS VII |
| Ukraine | Europe & Central Asia | Lower middle income | UABR51FL.DTA | 2007 | DHS V |
| Tanzania | Sub-Saharan Africa | Low income | TZBR7AFL.DTA | 2015 | DHS VII |
| Vietnam | East Asia & Pacific | Lower middle income | VNBR41FL.dta | 2002 | DHS IV |
| Yemen | Middle East & North Africa | Low income | YEBR61FL.DTA | 2013 | DHS VI |
| Zambia | Sub-Saharan Africa | Lower middle income | ZMBR61FL.DTA | 2013 | DHS VI |
| Zimbabwe | Sub-Saharan Africa | Low income | ZWBR71FL.DTA | 2015 | DHS VII |

*ANC1 timing data is not available for Bangladesh.
