# Supplementary material for: Inferring antenatal care visit timing in low- and middle-income countries: Methods to inform potential maternal vaccine coverage
Source: PLoS One. 2020 Aug 20;15(8):e0237718. doi: 10.1371/journal.pone.0237718 (PMC7446781; doi:10.1371/journal.pone.0237718)
Supplement: S2 Appendix — (DOCX) [file pone.0237718.s002.docx]

**Appendix 2: ANC1 timing by gestation month across countries [3]**

| **Country** | **Months** | | | | | | | | | | | | |
| --- | --- | --- | --- | --- | --- | --- | --- | --- | --- | --- | --- | --- | --- |
|  | **0** | **1** | **2** | **3** | **4** | **5** | **6** | **7** | **8** | **9** | **10** | **don't know** | **No respon**  **se** |
| Afghanistan | 0.03 | 6.12 | 19.59 | 21.39 | 17.32 | 10.72 | 9.44 | 6.36 | 3.62 | 1.22 | 0.05 | 4.13 |  |
| Albania | 1.32 | 38.35 | 33.95 | 18.27 | 4.1 | 1.17 | 0.59 | 0.34 | 0.29 | 0.93 |  | 0.68 |  |
| Angola | 0.3 | 5.03 | 13.76 | 25.24 | 25.49 | 15.79 | 9.25 | 2.85 | 0.94 | 0.57 | 0.06 | 0.72 |  |
| Armenia | 0.29 | 9.91 | 39.3 | 44.04 | 5.17 | 0.65 | 0.14 | 0.22 | 0.07 | 0.07 |  | 0.14 |  |
| Azerbaijan | 1.14 | 10.95 | 18.1 | 36.43 | 9.81 | 8.9 | 6.16 | 3.65 | 2.05 | 1.14 |  | 1.37 | 0.3 |
| Benin | 1.16 | 10.25 | 16.57 | 28.82 | 16.05 | 13.3 | 7.27 | 3.96 | 1.38 | 0.29 | 0.03 | 0.94 |  |
| Bolivia | 19.95 | 23.97 | 24.94 | 12.79 | 7.96 | 5.7 | 2.61 | 1.51 | 0.12 | 0.02 | 0.02 | 0.34 | 0.07 |
| Brazil | 1.66 | 21.74 | 26.26 | 25.15 | 13.39 | 5.61 | 3.81 | 1.28 | 0.69 | 0.09 |  | 0.33 |  |
| Burkina Faso | 0.03 | 1.09 | 6.95 | 34.32 | 25.1 | 16.67 | 10.31 | 4.27 | 0.99 | 0.15 |  | 0.11 |  |
| Burundi | 0.22 | 1.84 | 4.81 | 40.56 | 22.26 | 14.55 | 10.58 | 3.94 | 0.94 | 0.16 |  | 0.14 |  |
| Cambodia | 0 | 37.28 | 26.07 | 19.41 | 7.84 | 5.32 | 1.99 | 1.4 | 0.45 | 0.11 |  | 0.14 |  |
| Cameroon | 0.52 | 3.96 | 8.11 | 27.71 | 24.18 | 18.1 | 11.39 | 4.63 | 1.17 | 0.07 | 0.01 | 0.13 |  |
| Central A.R | 0 | 1.1 | 5.75 | 25.2 | 22.16 | 21.47 | 14.48 | 5.98 | 2.11 | 0.55 |  | 1.2 |  |
| Chad | 0.05 | 2.37 | 9.46 | 31.84 | 25.29 | 16.01 | 8.74 | 3.24 | 1.13 | 0.23 |  | 1.65 |  |
| Colombia | 2.16 | 29.64 | 27.6 | 19.69 | 10.11 | 5.3 | 3.02 | 1.34 | 0.58 | 0.33 |  | 0.22 |  |
| Comoros | 0.53 | 12.05 | 17.71 | 31.73 | 13.39 | 9.6 | 6.19 | 2.19 | 1.17 | 3.04 |  | 2.35 | 0.05 |
| Congo | 0.05 | 1.49 | 7.33 | 39.54 | 25.45 | 15.58 | 7.62 | 2.29 | 0.5 | 0.03 | 0.02 | 0.09 |  |
| Cote D Ivorie | 0.06 | 3.81 | 7.88 | 19.35 | 18.27 | 20.08 | 16.75 | 9.36 | 3.14 | 0.69 | 0.16 | 0.45 |  |
| DRC | 0.01 | 0.57 | 3.04 | 15.23 | 21.71 | 23.42 | 22.42 | 10.18 | 2.86 | 0.25 | 0.01 | 0.28 |  |
| Dom. Republic | 3.84 | 28.96 | 28.65 | 21.13 | 9.87 | 4.53 | 2.28 | 0.54 | 0.1 | 0.03 |  | 0.07 |  |
| Egypt | 0 | 21.19 | 49.97 | 14.24 | 7.76 | 3.07 | 1.43 | 1.18 | 0.68 | 0.26 |  | 0.21 |  |
| Ethiopia | 0.11 | 3.99 | 10.06 | 24.32 | 23.62 | 16.3 | 12.29 | 5.79 | 2.4 | 0.55 |  | 0.57 |  |
| Gabon | 0.5 | 4.64 | 10.4 | 43.12 | 21.51 | 10.4 | 7.05 | 1.55 | 0.39 | 0.13 |  | 0.24 | 0.08 |
| Gambia | 0.02 | 1.87 | 6.6 | 30.18 | 25.27 | 16.22 | 12.69 | 5.61 | 1.29 | 0.13 |  | 0.13 |  |
| Ghana | 0 | 9.85 | 19.87 | 35.74 | 19.05 | 8.67 | 4.35 | 1.83 | 0.38 | 0.22 |  | 0.05 |  |
| Guatemala | 0.57 | 18.03 | 26.28 | 26.5 | 13.5 | 7.83 | 4.64 | 1.93 | 0.69 | 0.02 |  | 0.01 |  |
| Guinea | 0.48 | 10.45 | 15.41 | 20.99 | 18.61 | 16.42 | 10.89 | 4.64 | 1.71 | 0.25 |  | 0.16 |  |
| Guyana | 0.39 | 5.52 | 10.2 | 31.38 | 19.23 | 15.01 | 8.25 | 3.96 | 1.56 | 0.19 |  | 2.01 | 2.27 |
| Haiti | 0.22 | 11.64 | 19.28 | 32.3 | 17.73 | 8.82 | 5.28 | 3.23 | 1.24 | 0.17 |  | 0.07 |  |
| Honduras | 0.44 | 24.58 | 31.54 | 21.95 | 10.74 | 5.14 | 3.14 | 1.47 | 0.83 | 0.11 |  | 0.05 | 0.01 |
| India | 0.2 | 6.6 | 21.04 | 42.18 | 14.26 | 7.97 | 2.61 | 1.66 | 1.2 | 1.74 | 0.2 | 0.32 |  |
| Indonesia | 1.35 | 36.59 | 25.01 | 18.18 | 8.54 | 4.84 | 2.52 | 1.43 | 0.63 | 0.5 | 0.02 | 0.4 |  |
| Jordan | 1.46 | 46.76 | 32.25 | 9.17 | 2.68 | 0.96 | 0.77 | 0.35 | 0.24 | 5.13 | 0.04 | 0.18 |  |
| Kazakhstan | 1.32 | 11.64 | 20.55 | 27.13 | 17.41 | 9.01 | 4.66 | 4.66 | 1.42 | 1.62 |  | 0.4 | 0.2 |
| Kenya | 0.14 | 1.76 | 4.29 | 12.22 | 20.01 | 24.55 | 22.87 | 10.75 | 2.73 | 0.48 |  | 0.19 |  |
| Kyrgyzstan | 0.13 | 10.38 | 36.31 | 36.08 | 10.9 | 3.5 | 1.46 | 0.71 | 0.19 | 0.13 |  | 0.19 |  |
| Lesotho | 0.24 | 6.34 | 11.71 | 23.99 | 20.84 | 16.16 | 11.87 | 6.1 | 1.45 | 0.93 | 0.04 | 0.32 |  |
| Liberia | 0.06 | 15.17 | 24.95 | 25.9 | 16.45 | 8.7 | 5.11 | 2.11 | 0.79 | 0.17 | 0.02 | 0.54 | 0.02 |
| Madagascar | 0.05 | 1.27 | 4.8 | 23.78 | 24.89 | 21.54 | 16.12 | 4.69 | 1.53 | 0.23 | 0.01 | 0.79 | 0.29 |
| Malawi | 0.05 | 0.48 | 2.24 | 23.3 | 30.17 | 21.28 | 16.15 | 4.84 | 0.94 | 0.36 | 0.03 | 0.17 |  |
| Maldives | 4.14 | 63.49 | 20.17 | 7.86 | 2.2 | 0.8 | 0.3 | 0.19 | 0.08 | 0.11 |  | 0.65 |  |
| Mali | 0.29 | 5.88 | 14.43 | 26.92 | 21.42 | 12.59 | 9.56 | 4.64 | 1.71 | 1.57 | 0.06 | 0.92 |  |
| Morocco | 2.05 | 25.36 | 18.53 | 28.37 | 10.9 | 4.42 | 3.98 | 2.63 | 2.08 | 1.57 |  | 0.1 |  |
| Mozambique | 0.06 | 0.51 | 2.33 | 12.42 | 23.77 | 27.61 | 23.16 | 7.18 | 1.96 | 0.4 | 0.01 | 0.59 |  |
| Myanmar | 0.15 | 6.07 | 12.5 | 27.44 | 18.59 | 15.64 | 9.57 | 6.34 | 2.51 | 0.57 |  | 0.63 |  |
| Namibia | 0.31 | 4.34 | 11.82 | 25.78 | 23.35 | 16.29 | 11.24 | 4.13 | 1.62 | 0.18 |  | 0.89 |  |
| Nepal | 0 | 7.62 | 14.65 | 47.04 | 18.11 | 7.19 | 3.3 | 1.23 | 0.61 | 0.24 |  |  |  |
| Nicaragua | 0.37 | 15.5 | 28.13 | 25.7 | 13.58 | 7.56 | 4.63 | 2.32 | 1.69 | 0.19 |  | 0.32 |  |
| Niger | 0 | 0.4 | 1.41 | 27.14 | 25.51 | 21.01 | 14.97 | 7.07 | 1.74 | 0.17 |  | 0.55 | 0.03 |
| Pakistan | 0.23 | 16.69 | 22.42 | 23.25 | 12.65 | 9.19 | 6.32 | 4.98 | 3.16 | 0.73 |  | 0.38 |  |
| Paraguay | 2.82 | 15.53 | 15.11 | 24.13 | 12.73 | 9.31 | 11.79 | 4.94 | 2.33 | 0.92 |  | 0.39 |  |
| Peru | 0.28 | 24.61 | 26.12 | 24.18 | 10.61 | 6.66 | 4.13 | 2.34 | 0.74 | 0.28 |  | 0.05 |  |
| Philippines | 0.53 | 10.82 | 18.78 | 38.75 | 15.06 | 10.04 | 3.59 | 1.44 | 0.53 | 0.29 | 0.03 | 0.13 |  |
| Moldova | 1.04 | 14.21 | 28.27 | 28.94 | 14.81 | 5.51 | 3.72 | 1.93 | 0.67 |  |  | 0.6 | 0.3 |
| Rwanda | 0.08 | 1.49 | 8.16 | 46.91 | 19.8 | 11.16 | 7.21 | 3.62 | 1.29 | 0.19 |  | 0.08 |  |
| Sao Tome Principe | 0.21 | 4.19 | 13.82 | 25.12 | 22.05 | 16.05 | 9.42 | 3.21 | 1.61 | 0.35 |  | 3.07 | 0.91 |
| Senegal | 0.29 | 5.8 | 16.07 | 40.49 | 15.55 | 10.39 | 5.56 | 2.18 | 0.7 | 0.25 |  | 2.71 |  |
| Sierra Leone | 0.02 | 2.85 | 11.5 | 31.02 | 27.7 | 14.79 | 8.37 | 1.84 | 0.37 | 0.31 | 0.01 | 1.02 | 0.19 |
| South Africa | 0.83 | 8.22 | 14.94 | 25.97 | 20.21 | 14.25 | 9.67 | 3.16 | 1.14 | 0.66 | 0.03 | 0.9 |  |
| Swaziland | 0.38 | 1.59 | 3.94 | 20.57 | 23.88 | 24.8 | 16.87 | 6.05 | 1.15 | 0.19 |  | 0.43 | 0.14 |
| Tajikistan | 0.08 | 5.67 | 29.14 | 38.44 | 13.7 | 7.12 | 2.57 | 1.93 | 0.56 | 0.36 |  | 0.43 |  |
| Timor-Leste | 0.85 | 14.22 | 23.02 | 28.68 | 18.66 | 7.26 | 3.44 | 0.97 | 0.94 | 1.46 | 0.17 | 0.33 |  |
| Togo | 0.11 | 2.79 | 4.88 | 19.74 | 22.86 | 22.75 | 16.73 | 7.26 | 2.51 | 0.26 |  | 0.11 |  |
| Turkey | 8.91 | 52.04 | 20.66 | 8.83 | 5 | 2.04 | 1.13 | 0.69 | 0.33 | 0.29 |  | 0.07 |  |
| Uganda | 0.16 | 2.25 | 6.47 | 21.49 | 29.09 | 19.48 | 14 | 5.08 | 1.58 | 0.25 | 0.03 | 0.12 |  |
| Ukraine | 0.18 | 9.84 | 38.18 | 33.85 | 9.38 | 3.96 | 1.75 | 0.74 | 0.28 | 0.09 |  | 1.1 | 0.64 |
| Tanzania | 0.01 | 0.55 | 3.64 | 18.7 | 25.3 | 23.74 | 18.47 | 7.51 | 1.69 | 0.23 |  | 0.14 |  |
| Uzbekistan | 0 | 9.09 | 30.17 | 37.38 | 12.77 | 5.88 | 2.27 | 1.02 | 0.08 | 0.08 |  | 1.25 |  |
| Vietnam | 0.7 | 9.11 | 15.41 | 39.05 | 12.52 | 7.79 | 7.44 | 4.99 | 2.63 | 0.26 |  | 0.09 |  |
| Yemen | 0.11 | 12.96 | 19.13 | 16.89 | 13.5 | 10.94 | 7.86 | 9.14 | 5.94 | 3.01 |  | 0.53 |  |
| Zambia | 0.02 | 0.49 | 3 | 20.41 | 32.01 | 24.07 | 14.02 | 4.48 | 0.92 | 0.15 | 0.01 | 0.41 |  |
| Zimbabwe | 0.28 | 1.73 | 5.03 | 33.28 | 19.69 | 17.75 | 12.8 | 6.35 | 2.49 | 0.46 |  | 0.13 |  |
