# Supplementary material for: Inferring antenatal care visit timing in low- and middle-income countries: Methods to inform potential maternal vaccine coverage
Source: PLoS One. 2020 Aug 20;15(8):e0237718. doi: 10.1371/journal.pone.0237718 (PMC7446781; doi:10.1371/journal.pone.0237718)
Supplement: S3 Appendix — (DOCX) [file pone.0237718.s003.docx]

**Appendix 3: ANC coverage and service availability and acceptance proxy estimates**

| **Country** | **iso3** | **ANC coverage rate (%) and data sources*** | | | | | | **Service availability and acceptance proxy***^+^* |
| --- | --- | --- | --- | --- | --- | --- | --- | --- |
|  |  | **Year** | **Source** | **ANC1** | **ANC2** | **ANC3** | **ANC4** |  |
| Afghanistan | AFG | 2015 | DHS 2015 | 58.60 | 45.00 | 31.40 | 17.80 | 0.51 |
| Albania | ALB | 2009 | DHS 2008–2009 | 97.30 | 87.13 | 76.97 | 66.80 | 0.90 |
| Angola | AGO | 2016 | DHS 2015–2016 | 81.60 | 74.87 | 68.13 | 61.40 | 0.82 |
| Armenia | ARM | 2016 | DHS 2015–2016 | 99.60 | 98.40 | 97.20 | 96.00 | 0.85 |
| Azerbaijan | AZE | 2011 | DHS 2011 | 91.70 | 83.17 | 74.63 | 66.10 | 0.63 |
| Bangladesh | BGD | 2014 | DHS 2014 | 63.90 | 53.00 | 42.10 | 31.20 | 0.68 |
| Benin | BEN | 2014 | MICS 2014 | 82.80 | 74.77 | 66.73 | 58.70 | 0.95 |
| Bolivia | BOL | 2012 | DHS 2012 | 90.10 | 85.17 | 80.23 | 75.30 | 0.82 |
| Burkina Faso | BFA | 2015 | Other NS 2015 | 92.80 | 77.60 | 62.40 | 47.20 | 0.78 |
| Burundi | BDI | 2017 | DHS 2016–2017 | 99.20 | 82.57 | 65.93 | 49.30 | 0.41 |
| Cambodia | KHM | 2014 | DHS 2014 | 95.30 | 88.73 | 82.17 | 75.60 | 0.83 |
| Cameroon | CMR | 2014 | MICS 2014 | 82.80 | 74.80 | 66.80 | 58.80 | 0.88 |
| Chad | TCD | 2015 | DHS 2014–2015 | 54.70 | 46.80 | 38.90 | 31.00 | 0.62 |
| Colombia | COL | 2015 | DHS 2015 | 97.20 | 94.77 | 92.33 | 89.90 | 0.96 |
| Comoros | COM | 2012 | DHS-MICS 2012 | 92.10 | 77.70 | 63.30 | 48.90 | 0.76 |
| Congo | COG | 2015 | MICS 2014–2015 | 93.50 | 88.67 | 83.83 | 79.00 | 0.90 |
| Côte d’Ivoire | CIV | 2016 | MICS 2016 | 93.20 | 79.23 | 65.27 | 51.30 | 0.73 |
| Dem.Rep.Congo | COD | 2014 | DHS 2013–2014 | 88.40 | 74.93 | 61.47 | 48.00 | 0.65 |
| Dom. Republic | DOM | 2014 | MICS 2014 | 98.00 | 96.30 | 94.60 | 92.90 | 0.93 |
| Egypt | EGY | 2014 | DHS 2014 | 90.30 | 87.80 | 85.30 | 82.80 | 0.76 |
| Ethiopia | ETH | 2016 | DHS 2016 | 62.40 | 52.20 | 42.00 | 31.80 | 0.60 |
| Gabon | GAB | 2012 | DHS 2012 | 94.70 | 89.00 | 83.30 | 77.60 | 0.89 |
| Gambia | GMB | 2013 | DHS 2013 | 86.20 | 83.33 | 80.47 | 77.60 | 0.93 |
| Ghana | GHA | 2014 | DHS 2014 | 90.50 | 89.43 | 88.37 | 87.30 | 0.94 |
| Guatemala | GTM | 2015 | DHS 2014–2015 | 91.30 | 89.60 | 87.90 | 86.20 | 0.81 |
| Guinea | GIN | 2016 | MICS 2016 | 84.30 | 73.07 | 61.83 | 50.60 | 0.64 |
| Guyana | GUY | 2014 | MICS 2014 | 90.70 | 89.37 | 88.03 | 86.70 | 0.90 |
| Haiti | HTI | 2017 | DHS (Prelim) 2016–2017 | 91.00 | 82.87 | 74.73 | 66.60 | 0.89 |
| Honduras | HND | 2012 | DHS 2011–2012 | 96.60 | 94.03 | 91.47 | 88.90 | 0.87 |
| India | IND | 2006 | NFHS 2005–2006 | 74.20 | 66.53 | 58.87 | 51.20 | 0.83 |
| Indonesia | IDN | 2013 | Other NS 2014 | 95.40 | 91.43 | 87.47 | 83.50 | 0.72 |
| Jordan | JOR | 2012 | DHS 2012 | 99.10 | 97.57 | 96.03 | 94.50 | 0.93 |
| Kazakhstan | KAZ | 2015 | MICS 2015 | 99.30 | 97.97 | 96.63 | 95.30 | 0.85 |
| Kenya | KEN | 2015 | MIS 2015 | 93.70 | 81.67 | 69.63 | 57.60 | 0.81 |
| Kyrgyzstan | KGZ | 2014 | MICS 2014 | 98.40 | 97.13 | 95.87 | 94.60 | 0.81 |
| Lesotho | LSO | 2014 | DHS 2014 | 95.20 | 88.27 | 81.33 | 74.40 | 0.84 |
| Liberia | LBR | 2013 | DHS 2013 | 95.90 | 89.97 | 84.03 | 78.10 | 0.89 |
| Madagascar | MDG | 2013 | ENSOMD 2012–2013 | 82.10 | 71.77 | 61.43 | 51.10 | 0.56 |
| Malawi | MWI | 2016 | DHS 2015–2016 | 94.80 | 80.07 | 65.33 | 50.60 | 0.74 |
| Maldives | MDV | 2009 | DHS 2009 | 99.10 | 94.43 | 89.77 | 85.10 | 0.97 |
| Mali | MLI | 2015 | MICS 2015 | 75.60 | 63.07 | 50.53 | 38.00 | 0.61 |
| Moldova | MDA | 2012 | MICS 2012 | 98.80 | 97.67 | 96.53 | 95.40 | 0.91 |
| Morocco | MAR | 2011 | ENPSF 2011 | 77.10 | 69.83 | 62.57 | 55.30 | 0.61 |
| Mozambique | MOZ | 2011 | DHS 2011 | 90.60 | 77.27 | 63.93 | 50.60 | 0.58 |
| Myanmar | MMR | 2016 | DHS 2015–2016 | 80.70 | 73.33 | 65.97 | 58.60 | 0.76 |
| Namibia | NAM | 2013 | DHS 2013 | 96.60 | 85.23 | 73.87 | 62.50 | 0.91 |
| Nepal | NPL | 2016 | DHS 2016 | 83.60 | 78.87 | 74.13 | 69.40 | 0.81 |
| Nicaragua | NIC | 2012 | Other NS 2012 | 94.70 | 92.40 | 90.10 | 87.80 | 0.86 |
| Niger | NER | 2012 | MICS 2012 | 82.80 | 67.87 | 52.93 | 38.00 | 0.57 |
| Nigeria | NGA | 2017 | MICS 2016-2017 | 65.80 | 60.23 | 54.67 | 49.10 | 0.77 |
| Pakistan | PAK | 2013 | DHS 2012–2013 | 73.10 | 60.93 | 48.77 | 36.60 | 0.72 |
| Paraguay | PRY | 2016 | MICS 2016 | 98.70 | 97.00 | 95.30 | 93.60 | 0.68 |
| Peru | PER | 2015 | DHS 2015 | 97.00 | 96.53 | 96.07 | 95.60 | 0.92 |
| Philippines | PHL | 2013 | DHS 2013 | 95.40 | 91.70 | 88.00 | 84.30 | 0.85 |
| Rwanda | RWA | 2015 | DHS 2014–2015 | 99.00 | 80.63 | 62.27 | 43.90 | 0.80 |
| Sao Tome Principe | STP | 2014 | MICS 2014 | 97.50 | 92.87 | 88.23 | 83.60 | 0.90 |
| Senegal | SEN | 2015 | DHS (Continuous) 2015 | 95.00 | 78.90 | 62.80 | 46.70 | 0.88 |
| Sierra Leone | SLE | 2013 | DHS 2013 | 97.10 | 90.07 | 83.03 | 76.00 | 0.89 |
| South Africa | ZAF | 2016 | DHS KIR 2016 | 93.70 | 87.63 | 81.57 | 75.50 | 0.97 |
| Swaziland | SWZ | 2014 | MICS 2014 | 98.50 | 91.03 | 83.57 | 76.10 | 0.87 |
| Tajikistan | TJK | 2012 | DHS 2012 | 78.80 | 70.03 | 61.27 | 52.50 | 0.85 |
| Tanzania | TZA | 2016 | DHS MIS 2015–2016 | 91.40 | 77.83 | 64.27 | 50.70 | 0.75 |
| Timor-Leste | TLS | 2010 | DHS 2009–2010 | 84.40 | 74.63 | 64.87 | 55.10 | 0.70 |
| Togo | TGO | 2014 | DHS 2013–2014 | 72.70 | 67.53 | 62.37 | 57.20 | 0.85 |
| Turkey | TUR | 2013 | DHS 2013 | 97.00 | 94.30 | 91.60 | 88.90 | 0.88 |
| Uganda | UGA | 2016 | DHS 2016 | 97.30 | 84.83 | 72.37 | 59.90 | 0.76 |
| Ukraine | UKR | 2012 | MICS 2012 | 98.60 | 94.80 | 91.00 | 87.20 | 0.49 |
| Yemen | YEM | 2013 | DHS 2013 | 59.80 | 48.23 | 36.67 | 25.10 | 0.65 |
| Zambia | ZMB | 2014 | DHS 2013–2014 | 95.70 | 82.30 | 68.90 | 55.50 | 0.84 |
| Zimbabwe | ZWE | 2015 | DHS 2015 | 93.30 | 87.43 | 81.57 | 75.70 | 0.87 |

*Compiled by UNICEF (Accessed June 2019) [9].

*^+^Service availability and acceptance proxy: Most DHS include data on ANC visitors who report receiving basic services during their ANC visit in their last pregnancy. The basic sets of ANC services included in the DHS are (1) informed of pregnancy complications, (2) weight measured, (3) height measured, (4) blood pressure taken, (5) urine sample taken, (6) blood sample taken, and (7) received iron supplementation. We consider the weighted average of the number of services received by ANC visitors as a proxy for the service availability and acceptance.*
