# Supplementary material for: Inferring antenatal care visit timing in low- and middle-income countries: Methods to inform potential maternal vaccine coverage
Source: PLoS One. 2020 Aug 20;15(8):e0237718. doi: 10.1371/journal.pone.0237718 (PMC7446781; doi:10.1371/journal.pone.0237718)
Supplement: S4 Appendix — (DOCX) [file pone.0237718.s004.docx]

**Appendix 4: Estimates of service availability and acceptance proxy by country used to adjust immunization coverage**
