# Supplementary material for: Inferring antenatal care visit timing in low- and middle-income countries: Methods to inform potential maternal vaccine coverage
Source: PLoS One. 2020 Aug 20;15(8):e0237718. doi: 10.1371/journal.pone.0237718 (PMC7446781; doi:10.1371/journal.pone.0237718)
Supplement: S5 Appendix — 2: ANC1 timing by gestation month by geographic region (Source: DHS, includes data from 2015–2018 only, N = 25). (DOCX) [file pone.0237718.s005.docx]

**Appendix 5.1: ANC1 timing by gestation month by geographic region (Source: DHS, includes data from 2010-2018 only, N=56)**

|  |  |
| --- | --- |
|  |  |
|  |  |

**Appendix 5.2: ANC1 timing by gestation month by geographic region (Source: DHS, includes data from 2015-2018 only, N=25)**

|  |  |
| --- | --- |
|  |  |
|  |  |
