# Supplementary material for: Inferring antenatal care visit timing in low- and middle-income countries: Methods to inform potential maternal vaccine coverage
Source: PLoS One. 2020 Aug 20;15(8):e0237718. doi: 10.1371/journal.pone.0237718 (PMC7446781; doi:10.1371/journal.pone.0237718)
Supplement: S6 Appendix — 2: Predicted RSV maternal immunization coverage at 24–36 weeks gestation by country (Includes data from 2015–2018 only, N = 25). (DOCX) [file pone.0237718.s006.docx]

**Appendix 6.1: Predicted RSV maternal immunization coverage at 24–36 weeks gestation by country (Includes data from 2010-2018 only, N=56)**

**Appendix 6.2: Predicted RSV maternal immunization coverage at 24–36 weeks gestation by country (Includes data from 2015-2018 only, N=25)**
