# Supplementary material for: Inferring antenatal care visit timing in low- and middle-income countries: Methods to inform potential maternal vaccine coverage
Source: PLoS One. 2020 Aug 20;15(8):e0237718. doi: 10.1371/journal.pone.0237718 (PMC7446781; doi:10.1371/journal.pone.0237718)
Supplement: S7 Appendix — 2 Comparison of RSV maternal immunization coverage predictions with estimates of ANC1, ANC4, and PAB (Includes data from 2015–2018 only). (DOCX) [file pone.0237718.s007.docx]

**Appendix 7.1 Comparison of RSV maternal immunization coverage predictions with estimates of ANC1, ANC4, and PAB (Includes data from 2010-2018 only).**

| **Panel a: Countries in sub-Saharan Africa (N=32)**   |
| --- |
| **Panel b: Countries outside sub-Saharan Africa (N=24)**   |

**Appendix 7.2 Comparison of RSV maternal immunization coverage predictions with estimates of ANC1, ANC4, and PAB (Includes data from 2015-2018 only).**

| **Panel a: Countries in sub-Saharan Africa (N=9)**   |
| --- |
| **Panel b: Countries outside sub-Saharan Africa (N=16)**   |
